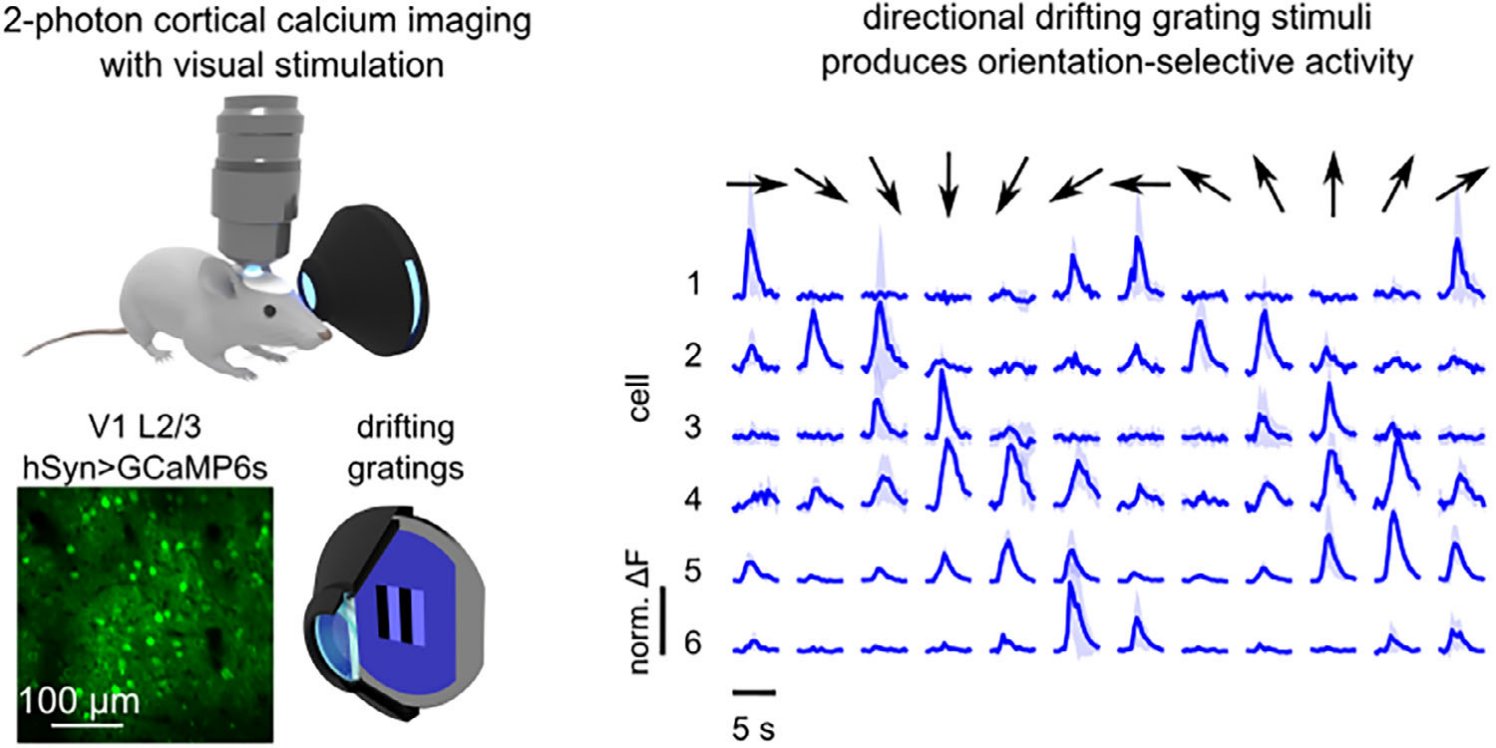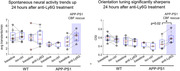# Increasing cerebral blood flow reduces neural activity derangement in mouse models of Alzheimer’s disease

**DOI:** 10.1002/alz.086922

**Published:** 2025-01-03

**Authors:** Rick T Zirkel, Matthew Isaacson, Michael Lamont, Nozomi Nishimura, Chris B Schaffer

**Affiliations:** ^1^ Cornell University, Ithaca, NY USA

## Abstract

**Background:**

Alzheimer’s disease (AD) is characterized by progressive, irreversible neurodegeneration, leading to memory loss and cognitive decline. In mouse models of AD, global decreases in cerebral blood flow (CBF) are brought on by the plugging of capillaries by arrested neutrophils, and the administration of the neutrophil‐specific antibody against Ly6G (anti‐Ly6G) reduces these capillary stalls in minutes and improves cognitive function within hours. This suggests that at least some aspects of neural activity impairment are reversible, but the mechanism of this recovery – and what specific neural activity is normalized – is not yet known. In agreement with prior studies, we found orientation tuning selectivity to drifting gratings in primary visual cortex neurons to be broadened in mouse models of AD. Here, we hypothesized that the impaired neural response can be modified by blood flow improvement with anti‐Ly6G treatment (4 mg/kg) in the APP/PS1 mouse model of AD.

**Method:**

We transfected neurons in layer 2/3 of the primary visual cortex (V1) of mice with a fluorescent calcium indicator using AAV9 vectors (pAAV.Syn.GCaMP6s.WPRE.SV40, 10^12 vg/mL). We also injected the fluorescent labels methoxy‐X04 to detect amyloid plaques and Texas‐red‐dextran in the vasculature to detect blocked capillaries. Drifting grating visual stimuli were presented to anesthetized mice with using MouseGoggles during recording with two‐photon microscopy before and one day after anti‐Ly6G or isotype control antibody administration (Fig. 1).

**Result:**

One day after anti‐Ly6G administration to reduce stalls and increase CBF, we observed a significant sharpening of orientation tuning relative to baseline in the same animals, as well as a trend toward increased spontaneous activity in V1 neurons (Fig. 2). Such normalization of neural activity patterns likely underlies the improved cognitive function that is seen within hours of blood flow increase in AD mouse models.

**Conclusion:**

Our data suggests some aspects of the neural and behavioral deficits in AD are acutely recoverable by increasing CBF. Such recovery demonstrates a promising avenue for future therapeutic targets to combat the symptoms of AD in humans.